# Enhanced Surface-Engineering Properties of Nanocrystalline Ceramic Coatings for Thermal Spray Applications

**DOI:** 10.3390/ma19091760

**Published:** 2026-04-25

**Authors:** George V. Theodorakopoulos, Nikolaos P. Petsas, Evangelos Kouvelos, Fotios K. Katsaros, George Em. Romanos

**Affiliations:** 1 Institute of Nanoscience and Nanotechnology, National Center for Scientific Research “Demokritos”, Agia Paraskevi, 15341 Athens, Greece; v.kouvelos@inn.demokritos.gr (E.K.); f.katsaros@inn.demokritos.gr (F.K.K.); 2 School of Chemical Engineering, National Technical University of Athens, Zografou Campus, 9 Iroon Polytechniou Street, Zografou, 15772 Athens, Greece

**Keywords:** HVOF, nanophase powder, WC, decarburization, aluminum addition, microhardness, pin-on-disk, counterbody wear

## Abstract

**Highlights:**

- Powder particle size strongly controls decarburization and nanostructure retention.
- Larger nanophase particles help preserve WC while limiting degradation during HVOF process.
- Harder coatings increase counterbody wear despite negligible coating wear.
- Al additions reduce Co_3_W_3_C formation and increase coating microhardness.
- Al offers microstructural benefits but raises friction, requiring application-specific tuning.

**Abstract:**

Wear remains a dominant cause of performance loss and premature failure in mechanical components, motivating the development of environmentally benign surface-engineering solutions. Among thermal spray systems, high-velocity oxy-fuel (HVOF)-sprayed WC-Co coatings are widely applied under severe wear conditions. The development of nanophase coatings offers the potential for enhanced mechanical performance. However, retaining the nanostructure and limiting decarburization during deposition remain key challenges. In this study, nanophase WC-12Co feedstocks with two particle size ranges, together with Al-modified nanophase powders, were used to deposit coatings under optimized HVOF spraying conditions (spray distance 200 mm, reduced O_2_/fuel ratio, and high particle velocity) and were benchmarked against a conventional WC-12Co (12 wt.% Co) coating. The coatings were characterized in terms of microstructure and phase constitution (OM, SEM/EDS, XRD) as well as thickness, porosity (0.5–3.6%), adhesion strength (up to 65 MPa), and microhardness (~1040–1210 HV). Tribological behavior was assessed by ASTM G99 pin-on-disk testing and counterbody wear was quantified via geometric volume loss estimations. The use of larger nanophase particles enabled effective nanostructure retention with limited decarburization, whereas reducing particle size intensified decarburization, promoting increased W_2_C formation, and markedly reduced coating cohesion, despite lower porosity and higher hardness. Aluminum additions enhanced coating microhardness and suppressed Co_3_W_3_C formation, indicating improved phase stability with minimal additional decarburization. Although coating wear remained negligible for all systems, Al-containing coatings exhibited increased friction (up to 35%) and significantly higher counterbody wear (up to sevenfold) compared to the Al-free nanophase coating, which was found to correlate with coating microhardness. Overall, the results demonstrate that optimizing nanophase WC-Co coatings requires balancing competing mechanisms between microstructural stability, cohesive integrity, and tribological response, highlighting the critical role of feedstock design in tailoring coating performance.

## 1. Introduction

The integrity and reliability of metallic components employed in mechanical and industrial systems are strongly influenced by surface-related degradation phenomena, most notably wear, erosion, and corrosion [1,2]. These processes progressively modify surface morphology and degrade material properties, leading to reduced operational efficiency, increased maintenance demands, and ultimately premature component failure [2]. The economic impact associated with wear- and corrosion-induced damage is substantial, accounting for a significant share of global industrial losses annually [3,4]. Nevertheless, a considerable proportion of these losses could be mitigated through the implementation of advanced surface engineering strategies and appropriate and effective material protection technologies [1,2,3,5]. Beyond the economic dimension, increasingly stringent environmental regulations have accelerated the transition away from traditional surface treatment methods, such as hard chromium electroplating, due to their toxicity and associated environmental burden [6,7,8,9,10]. This shift has intensified research efforts toward environmentally benign and technologically robust alternatives capable of delivering equivalent or superior performance [8,9]. Within this context, thermal spray technologies have emerged as a versatile and effective approach for enhancing the surface properties of engineering components [1,2,11,12]. By enabling the deposition of compact, well-adhered coatings with tailored microstructures, thermal spraying provides substantial improvements in resistance to wear, corrosion, and high-temperature degradation [1,11,12]. Consequently, thermal spray coatings are now widely regarded as a key solution for extending component service life while simultaneously addressing the economic and environmental challenges faced by modern industry [11,13,14].

Among the broad range of material systems processed by thermal spray technologies, carbide-based cermets, and particularly tungsten carbide with cobalt (WC-Co) as a metallic binder, have attracted sustained scientific and industrial interest due to their outstanding wear resistance. WC-Co coatings are typically recommended for operating temperatures below 500 °C, as exposure to higher temperatures promotes the formation of brittle secondary phases that degrade coating performance and reduce wear resistance [15]. The final properties of WC-based coatings are strongly governed by feedstock characteristics, including the powder production route, WC grain size, and spraying parameters. Such coatings are typically applied in environments requiring resistance to mechanical erosion, abrasion, fretting, sliding, and impact loading. A higher cobalt content in the binder phase enhances coating toughness, whereas alloying additions such as chromium have been shown to improve corrosion resistance in aggressive environments. Despite the well-established performance of conventional WC-Co coatings, continuous efforts to further enhance durability and functional performance have driven increasing interest in coatings produced from nanocrystalline feedstock. In particular, significant attention has been devoted to understanding the influence of nanoscale microstructures on the mechanical and tribological behavior of thermally sprayed WC-Co coatings [16,17,18]. High-velocity oxy-fuel (HVOF) spraying is generally regarded as the preferred deposition technique for such materials, owing to its relatively lower flame temperatures combined with exceptionally high particle velocities achieved during spraying, which favor dense coatings with limited oxidation. Among available coating technologies, including physical vapor deposition (PVD) and chemical vapor deposition (CVD), thermal spray techniques, particularly HVOF, offer distinct advantages for the deposition of thick, wear-resistant WC-Co coatings, including high deposition rates, strong mechanical interlocking, high adhesion strength, and the ability to process carbide-based cermets with limited oxidation. In contrast, PVD coatings are typically limited to thin films and are less suitable for applications requiring high load-bearing capacity, thick coatings, and resistance to severe wear conditions [19,20].

However, the literature reports conflicting conclusions regarding the anticipated benefits of nanocrystalline WC-Co coatings, especially with respect to improvements in wear resistance compared to their conventional counterparts. While several studies report enhanced hardness and improved tribological performance, others indicate that nanostructured coatings may, under certain conditions, exhibit inferior wear behavior. This apparent discrepancy has been primarily attributed to microstructural degradation phenomena occurring during deposition. More specifically, nanocrystalline WC-Co coatings may exhibit pull-out of fine WC grains during sliding contact with the counterbody material, as well as partial decomposition of WC during spraying. Such decomposition, typically associated with decarburization, leads to the formation of undesirable brittle phases such as W_2_C, WC_1−x_, and metallic W. Decarburization and phase transformation mechanisms during thermal spraying, including WC dissolution and formation of secondary carbides, are therefore critical factors influencing coating performance and are discussed in detail in the following sections. In parallel, reactions between dissolved WC and the cobalt binder may reduce the fraction of the hard phase and promote the formation of complex secondary carbides, including Co_3_W_3_C and Co_6_W_6_C, further deteriorating wear performance [16,21]. These degradation pathways are closely linked to microstructural evolution during deposition, which brings into focus one of the principal challenges associated with processing nanostructured feedstocks: the preservation of nanoscale features within the deposited coating. A major challenge in the thermal spraying of nanostructured materials therefore lies in preserving the nanoscale grain size of the original feedstock within the deposited coating [22]. During deposition, particles are subjected to intense thermal and kinetic energy, which can induce grain growth and coarsening, phase transformations, or partial melting, and decarburization thereby compromising the initial nanostructure [22]. Nevertheless, when deposition parameters are appropriately optimized, nanostructured feedstocks can yield coatings with refined microstructures and enhanced interlamellar cohesion [23]. Such features are generally associated with improved hardness and enhanced resistance to wear and corrosion compared with coatings produced from conventional, coarser-grained powders [24].

Previous studies on HVOF-sprayed nanostructured WC-Co coatings produced via high-energy ball-milling routes have demonstrated the feasibility of depositing dense, well-adherent nanostructured coatings with crystallite sizes below 30 nm [25,26]. In these studies, aluminum was introduced as an alloying addition with the objective of improving coating cohesion and tribological performance. The presence of aluminum was reported to enhance carbide particle distribution, suppress the formation of brittle W_2_C phases, and improve wear resistance relative to both nanostructured and conventional coatings [25]. Complementary work examining corrosion behavior likewise investigated the influence of Al alloying on nanostructured WC-Co coatings, revealing its effect on electrochemical response and interfacial reactivity [26]. Despite these advances, several critical aspects remain insufficiently explored. In particular, the role of feedstock particle size in governing decarburization phenomena, nanostructure retention, and phase stability during HVOF spraying has not been systematically clarified. Moreover, the combined influence of particle size and compositional modifications on the resulting tribological performance of nanophase coatings remains inadequately understood. Furthermore, while aluminum additions have been associated with improved wear performance, their influence on coating cohesion, adhesion integrity, and counterbody wear aggressiveness under severe sliding conditions remains inadequately understood. In particular, the potential trade-off between increasing coating hardness and exacerbating wear damage to the opposing material has not been quantitatively assessed. The novelty of the present study lies in the combined investigation of feedstock particle size and aluminum addition, together with the explicit evaluation of counterbody wear in relation to coating microstructure and tribological performance.

In this context, the present study provides a systematic evaluation of their combined effects on the microstructural integrity and surface-engineering performance of HVOF-sprayed nanocrystalline WC-Co coatings. Nanophase feedstocks with two distinct particle size ranges, together with Al-modified nanophase powders, were investigated and directly benchmarked against a conventional industrial WC-Co coating. The resulting coatings were characterized in terms of microstructure, phase constitution, porosity, adhesion strength, deposition efficiency, and microhardness, with particular emphasis placed on assessing nanostructure retention after deposition. In addition, tribological performance was evaluated through long-duration pin-on-disk testing to examine frictional behavior and wear response. Through this integrated approach, the present study aims to elucidate the relationships between processing conditions, microstructural evolution, and resulting properties in nanophase WC-Co coatings, with particular emphasis on tribological performance and wear behavior, and to identify the critical trade-offs required to achieve an optimal balance among microstructural stability, mechanical integrity, and tribological performance.

## 2. Materials and Methods

### 2.1. Feedstock Materials

The nanophase powders (88 wt.% WC-12 wt.% Co) are collectively denoted as NP. Two particle-size variants were employed in the present study: a coarse fraction, labeled NP-L (−75 + 38 μm) and a finer fraction, labeled NP-S (−38 + 15 μm). Apart from particle size, all other characteristics of these powders were identical, including a reported WC grain size of approximately 13 nm, confirming their nanocrystalline nature. Aluminum-modified nanophase powders were also investigated and are designated NP-2Al and NP-3Al, corresponding to 2 and 3 wt.% aluminum additions, respectively. It should be noted that all nanophase powders employed in this work were supplied by the same manufacturer (MBN srl, Treviso, Italy) and were produced via high-energy ball milling. Consequently, they exhibit comparable morphology and processing history. Furthermore, the Al-containing powders share the same particle size range as NP-L (−75 + 38 μm), thereby excluding particle size as a variable in assessing the effect of aluminum addition. For comparison, a conventional powder, denoted as CP, was also examined. This feedstock has a chemical composition of 82 wt.% W, 5.4 wt.% C, and 12 wt.% Co, and a nominal particle size range of −50 + 10 μm. Its particle size distribution shows no residue in the +230 mesh (63 μm) and +270 mesh (53 μm) fractions, while a small fraction (5 wt.%) is retained in the +325 mesh (45 μm) range. These compositional and granulometric differences clearly distinguish the nanophase powders from the conventional feedstock and form the basis for the comparative investigation conducted in this study.

### 2.2. Coating Deposition (HVOF Spraying)

**Figure 1:**
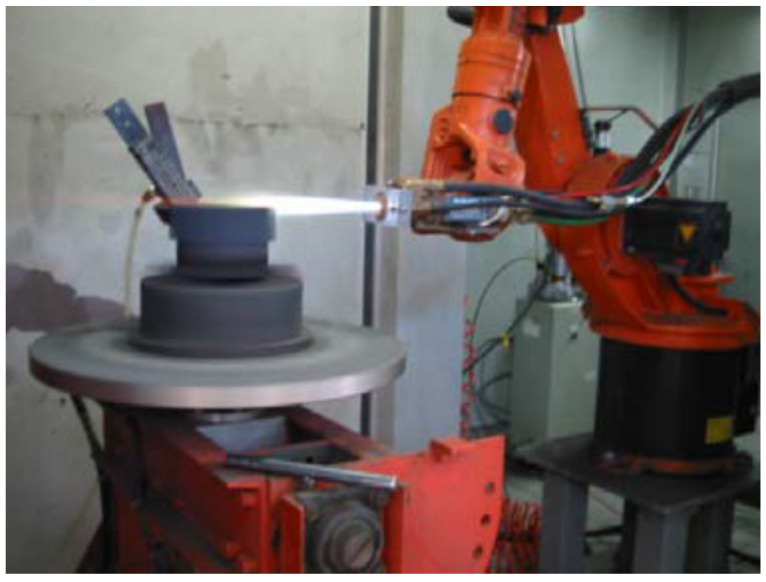
HVOF thermal spraying process during operation, showing the high-velocity particle jet and its interaction with the rotating substrate during coating deposition.

A representative image of the HVOF spraying system during operation is shown in Figure 1, illustrating the coating deposition process, including the spray gun, high-velocity particle jet, and interaction with the rotating substrate.

Based on previous qualitative and quantitative studies on the influence of spraying parameters on coating properties [27], an optimized combination of spraying parameters was adopted, as summarized in Table 1. The spraying parameters were defined to balance particle temperature and velocity, aiming to minimize decarburization while ensuring sufficient particle deformation upon impact. In particular, a relatively high combustion gas flow rate combined with a reduced oxygen-to-fuel ratio was employed to decrease particle residence time in the flame and limit WC degradation. The chosen particle size ranges were designed to systematically investigate the effect of thermal exposure and heat transfer on nanostructure retention during spraying. Similarly, the aluminum content (2–3 wt.%) was selected based on the literature evidence indicating its effectiveness in improving coating cohesion and suppressing undesirable phase formation without significantly altering feedstock morphology.

All other spraying parameters were kept constant throughout the experiments. In addition, an air-cooling system using atmospheric air was employed to prevent overheating of the substrate. The fixed spraying conditions included a total combustion gas flow rate of 760 L/min, an argon carrier gas flow rate of 14.5 L/min, 20 spray passes, and air cooling at a pressure of 40 psi.

### 2.3. Microstructural and Phase Characterization

Microstructural characterization was conducted using a Jeol JSM-6300 Scanning Electron Microscope (Tokyo, Japan). Secondary electron (SE) imaging was primarily used for morphological observations, while backscattered electron (BSE) imaging was applied where appropriate to provide compositional contrast. Powders and coating cross-sections were examined using a Leica Q550MW Optical Microscope (Wetzlar, Germany) coupled with computer-assisted image analysis to determine coating thickness and porosity. Quantitative measurements were performed on polished cross-sectional micrographs. Coating thickness was evaluated from fifteen measurements across five different regions of each specimen, in accordance with the ASTM B487-85 standard [28]. Porosity was determined by quantitative analysis of the same micrographs, with measurements performed on representative coating areas to ensure statistical reliability. XRD diffraction patterns were recorded using a Siemens D5000 X-ray diffractometer (Munich, Germany) equipped with CuKα1 radiation (λ = 1.5405 Å) and a Ni filter, operating at 30 kV and 10 mA. Diffraction data were collected over a 2θ range of 15–90° at a scanning rate of 0.02°/s.

### 2.4. Mechanical Properties

Microhardness measurements (HV) were carried out using a Buehler Micromet 2100 Microhardness Tester (Uzwil, Switzerland) equipped with an integrated optical microscope. Indentations were performed under a load of 300 g (HV_0.3_) with a dwell time of 10 s. To ensure statistical reliability, fourteen measurements were taken at different locations on each coating. All coatings exhibited thicknesses greater than 50 μm, while the corresponding indentation depth (~2–4 μm), estimated based on Vickers indentation geometry and the measured hardness values, is significantly smaller, thereby ensuring negligible substrate influence on the measured hardness values. Adhesion strength was experimentally determined using a pneumatic pull-off adhesion testing system, the Elcometer 110 PATTI Adhesion Tester (Manchester, UK), following the ASTM D4541-95 standard [29].

### 2.5. Tribological Testing

Tribological performance, particularly wear resistance, represents a key property of WC-12Co coatings in both scientific and industrial contexts. Previous studies have shown that HVOF-sprayed WC-12Co coatings exhibit excellent wear resistance [30,31]. To assess the antifriction properties of the coatings, tribological tests were performed using a pin-on-disk tribometer (CSM High Temperature POD Tribometer, CSEM Instruments SA, Peseux, Switzerland), in accordance with the ASTM G99-90 standard [32]. The wear tests were conducted using an Al_2_O_3_ counterbody ball (6 mm in diameter, approximately 1900 HV_0.3_) at a rotational speed of 382 rpm and a sliding speed of 300 mm/s, with a wear track radius of 7.5 mm under a constant normal load of 10 N. All experiments were carried out under dry sliding conditions at room temperature (25 °C) and 65% relative humidity. The selected testing conditions (10 N load, 300 mm/s sliding speed, and 150,000 cycles) were intentionally chosen to represent severe dry sliding conditions, ensuring measurable wear effects and enabling reliable comparison among coatings with very high wear resistance, while approaching the operational limits of conventional tribometers. The tests were conducted to evaluate the average coefficient of friction (μ = F_F_/F_N_, where F_F_ is the friction force and F_N_ is the normal force), as well as the wear of both the coating and the counterbody in terms of volume loss. To determine the volumetric wear of the coatings after completion of the experiments, a surface profilometer Hommel Tester T1000 (HOMMEL ETAMIC—JENOPTIK Industrial Metrology Germany GmbH, Villingen-Schwenningen, Germany) was employed.

## 3. Results

### 3.1. Powder Morphology

**Figure 2:**
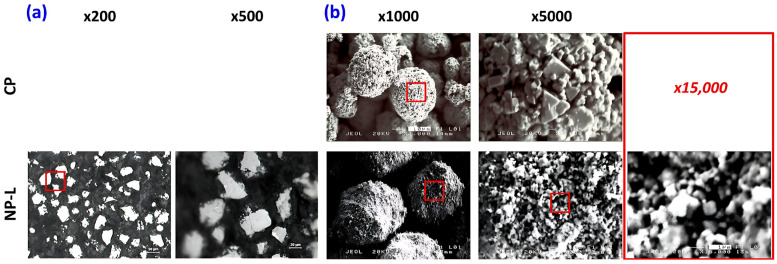
(**a**) OM and (**b**) SEM micrographs showing the morphology of CP and NP-L powders. Red boxes in the lower-magnification images indicate the exact regions examined at higher magnifications.

The WC grain size reported for the nanophase powder is provided by the manufacturer and was determined from XRD peak broadening using the Scherrer equation, yielding a value of approximately 13 nm for the nanocrystalline material [33,34]. The particle size of the nanophase powder was intentionally selected to be larger than that of the conventional powder in order to promote retention of the nanostructure after spraying. Accordingly, the nominal particle size range of the nanophase powder (NP-L) was −75 + 38 μm, whereas that of the conventional powder was −50 + 10 μm. The structure and morphology of the two powders are illustrated in the micrographs shown in Figure 2, obtained using optical and scanning electron microscopy.

The micrographs indicate that the particles of the conventional powder possess a predominantly spheroidal with rough surfaces characterized by numerous asperities/protrusions arising from exposed WC grains. As reported by the powder manufacturer, this material is produced via agglomeration and sintering, and the observed morphology is typical of this widely applied processing route for WC-Co powders [35]. Such agglomerated particles consist of micrometer-scale WC grains bonded by a cobalt matrix, a structure that is known to promote good flowability during spraying. By comparison, the nanophase powder was synthesized using high-energy ball milling, a technique commonly employed for the large-scale production of nanostructured powders under controlled, often vacuum, conditions, owing to its effectiveness in refining grain size, promoting homogeneous phase distribution, and enabling scalable feedstock manufacturing for thermal spray applications [36,37,38,39,40]. As shown in Figure 2b at ×1000 magnification, the NP-L powder exhibits a larger particle size than the CP powder. The particles display relatively rounded edges and are composed of agglomerated WC grains in the nanometer size range, as illustrated in Figure 2b at ×15,000 magnification, a feature that is expected to influence both melting behavior during spraying and the resulting coating microstructure.

### 3.2. Structure, Morphology, and Mechanical Properties of HVOF-Sprayed WC-12Co Coatings

The produced nanophase coating exhibits an optimized combination of properties, as summarized in Table 2, which also includes the corresponding properties of the conventional coating for reference. The nanophase coating shows a slight improvement in deposition efficiency, expressed as thickness per pass, compared with the conventional coating. Adhesion strength remains at similarly high levels for both coatings, a feature commonly observed in coatings deposited by the HVOF technique [41].

**Figure 3:**
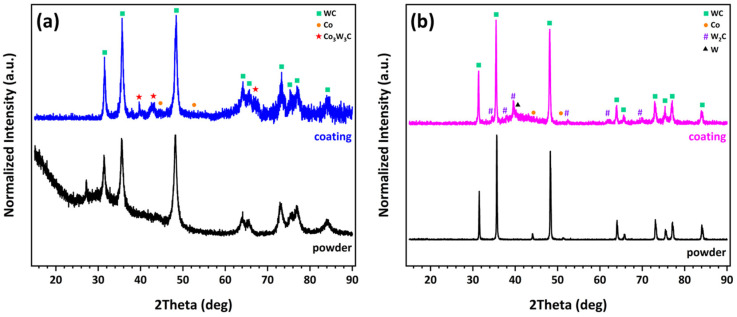
XRD patterns of (**a**) NP-L and (**b**) CP powders and their corresponding HVOF-sprayed coatings, with identified crystalline phases indicated.

Porosity is higher in the coating produced from the nanophase powder, with values increasing from 0.5% in the CP coating to 3.6% in the NP-L coating (Table 2), corresponding to an approximately sevenfold increase, with the larger powder particle size of the nanophase feedstock being the most probable contributing factor relative to the conventional powder. In addition, the higher kinetic energy of the sprayed particles, resulting from the increased total combustion gas flow rate, employed to reduce particle residence time in the flame, combined with the lower oxygen-to-fuel ratio, which decreases flame temperature and promotes a non-oxidizing environment, favors only partial melting or plastic deformation of the particles. Under these spraying conditions, the flattening of partially molten particles upon impact with the substrate is less effective, leading to increased porosity in the resulting coating. This behavior is consistent with several literature reports on HVOF-sprayed WC-Co coatings [35,42,43]. With respect to microhardness, the coating produced from the nanophase powder exhibits a lower value compared with the conventional coating. The higher porosity is a primary contributing factor, as these two parameters are correlated through the following relationship:

(1) $$\frac{H}{H_{0}}=e^{-\alpha \times P}$$

where H denotes the coating hardness (MPa), H_0_ the hardness of the fully dense (non-porous) coating material (MPa), P the porosity (-), and α an empirical constant. According to this relationship, an increase in porosity results in a reduction in microhardness, a trend that has also been reported in several previous studies [44,45,46]. In addition, partial loss of the WC phase during spraying of the NP-L powder, due to its dissolution into the cobalt binder and the subsequent formation of the Co_3_W_3_C phase (Figure 3a), further contributes to the decrease in coating microhardness. Although Co_3_W_3_C is harder than the original metallic Co phase, the reduction in the fraction of the WC hard phase has a more pronounced effect on the overall hardness of the coating [41,42].

**Figure 4:**
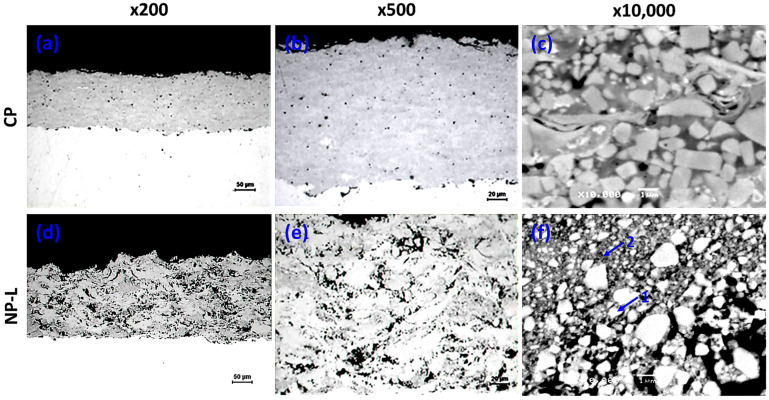
(**a**–**c**) Cross-sectional OM and SEM micrographs of the CP coating and (**d**–**f**) those of the NP-L coating deposited under optimized spraying conditions, shown at increasing magnifications; (**f**) points 1 and 2 denote EDS analysis locations.

Conversely, the formation of the hard and brittle W_2_C phase in the conventional coating (Figure 3b) promotes higher microhardness values in this case. It should also be noted that a reduction in grain size generally leads to an increase in hardness [44,47,48], an effect commonly described by the Hall–Petch relationship:

(2) $$H=H_{0}+k_{H}\times d^{-1/2}$$

where H represents the coating hardness (MPa), while H_0_ (MPa) and k_H_ are material-dependent constants and d is the average grain diameter (mm) [42,46,49]. Based on the above considerations, it can be concluded that the combined effects of increased porosity and the formation of the Co_3_W_3_C phase, accompanied by a reduction in WC content in the nanophase coating, exert a stronger influence on decreasing microhardness than the expected hardening effect associated with the reduced WC grain size. Subsequently, OM and SEM micrographs of the conventional coating (Figure 4a–c) and of the optimized coating produced from NP-L powder (Figure 4d–f) are presented.

**Figure 5:**
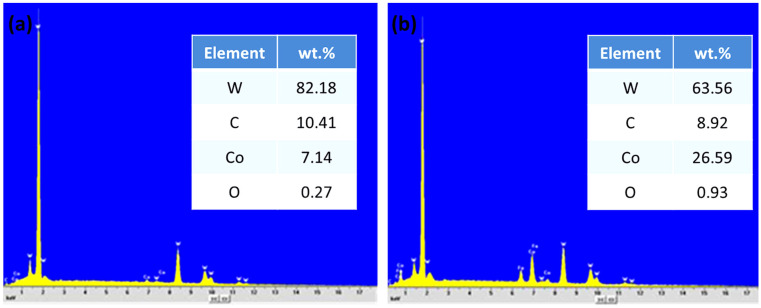
EDS spectra at (**a**) location (1) and (**b**) location (2) indicated in Figure 4f, with corresponding elemental compositions (wt.%).

Both coatings exhibit very good adhesion to the substrate. No horizontal or vertical cracks are observed, while the micrographs clearly reveal a higher level of porosity in the coating produced from the nanophase powder. Furthermore, Figure 4f shows the presence of numerous grains with sizes of only a few nanometers, along with the simultaneous occurrence of grains in the range of 1–2 μm. These larger grains are estimated to originate from agglomeration and grain growth during spraying. During the HVOF process, particles undergo partial melting and may coalesce to form agglomerates, resulting in the observed bimodal size distribution characterized by the coexistence of nanometer-sized grains and micrometer-scale agglomerates. For this reason, the coating can be appropriately classified as nanophase. EDS point analyses performed at location 1 (bright region) in Figure 4f revealed a high W content (Figure 5a), indicating that the bright regions correspond to WC grains. In contrast, analyses at location 2 (gray region) detected the presence of W, C, and Co (Figure 5b).

This region can therefore be attributed to the binder phase, which is inferred to have transformed into the Co_3_W_3_C phase after spraying, in agreement with the XRD results (Figure 3a). It has been reported that during powder milling, WC grains undergo fragmentation and become incorporated into the binder phase [47]. As a result, the binder phase is mixed with fragmented WC grains, and a pure crystalline Co phase is rarely present in the powder. Consequently, the effective Co content of the binder phase is reduced in nanocrystalline coatings. In addition, the determination of elemental concentrations is influenced by the spatial resolution limitations of the EDS electron beam. Given that both the binder phase and WC grains are extremely fine in the coating, it is difficult to identify regions consisting exclusively of either the binder phase or WC grains. As a result, the chemical composition measured in the binder phase typically includes contributions from WC, and vice versa.

XRD analysis of the nanophase powder and the corresponding coating indicates that the initial powder structure is largely preserved in the coating, as evidenced by the presence of WC diffraction peaks. The metallic Co phase is not detected in the coating; instead, an amorphous or nanocrystalline Co_3_W_3_C phase is observed. As discussed previously, this phase forms during spraying because of WC dissolution into the cobalt binder under the high temperatures developed in the flame. The formation of Co_3_W_3_C through the following reaction has been reported in several studies [50,51]:

3Co + 3WC + O_2_ ⟷ Co_3_W_3_C + 2CO (3)

As a WC-12Co powder particle enters the hot gas stream of the spraying flame, its temperature rises and the metallic cobalt phase begins to melt after a relatively short exposure time, given that pure Co melts at approximately 1495 °C. Once the Co is molten, WC grains start to dissolve rapidly into the melt. As the particle temperature continues to increase during spraying, progressively larger amounts of WC are dissolved [52]. Subsequently, carbon is depleted from the melt, either through reactions with oxygen at the melt/gas interface or via oxygen diffusion into the outer region of the molten particle, leading to the formation of CO. Carbon depletion is therefore largely confined to the peripheral region of the particle, with the affected depth depending on carbon and oxygen transport phenomena as well as reaction kinetics. However, this localized carbon loss further promotes dissolution of WC grains in the particle periphery, as the system tends to reestablish equilibrium at the WC/melt interface. Upon impact with the substrate, the particle undergoes flattening, rapid cooling, and solidification. As a result, WC grains in the outer peripheral region of the particle become less angular, as observed in Figure 4f, due to partial dissolution. According to the literature, this region also exhibits a reduced WC volume fraction compared with the particle core [52].

With regard to the metallic matrix, following the dissolution of WC grains, the melt consists of a liquid Co(W,C) phase. Upon impact with the substrate, cooling and solidification occur almost instantaneously, whereas the kinetics of formation of the complex crystalline Co_3_W_3_C phase are comparatively slower. As a result, the liquid phase solidifies into an amorphous structure. The subsequent development of nano-crystallinity can be attributed to partial crystallization of this amorphous phase induced by repeated reheating during successive spray gun passes in the coating buildup process. The WC and Co crystallite sizes in the nanophase powder were estimated using the Scherrer equation to be in the ranges of 11–19 nm and 15–20 nm, respectively. These values are in good agreement with the nominal grain size reported by the manufacturer (13 nm). For the coating, crystallite size estimation was performed using the three main WC diffraction peaks at 31.5°, 35.5°, and 48°, yielding values in the range of 18–45 nm. It should be noted that instrumental peak broadening and the contribution of internal microstrains, both of which can also cause peak broadening, were not considered in these calculations [42,53]. Consequently, the actual grain size may be slightly larger than the estimated values; however, it remains below 100 nm in all cases. Overall, a very satisfactory retention of the nanostructure is achieved, with only a modest increase in WC grain size relative to the initial powder.

In the case of the conventional coating (Figure 3b), in addition to the WC diffraction peaks, peaks corresponding to the W_2_C phase are also observed, which are attributed to decarburization phenomena. As discussed previously, the formation of this hard and brittle phase provides an explanation for the higher microhardness measured in the conventional coating. Furthermore, a small amount of metallic W is detected, also resulting from decarburization. The Co peaks initially present in the powder are no longer observed in the coating. In the 42–48° diffraction range, the development of an amorphous phase is evident, a feature that has been widely reported in the literature [51,54]. The main decarburization reactions that typically occur during the thermal spraying of WC-Co are summarized below [50,51]:

2WC + O_2_ ⟷ W_2_C + CO_2_ (4)

W_2_C + ½O_2_ ⟷ W_2_(CO) (5)

W_2_(CO) ⟷ 2W + CO (6)

These reactions occur primarily in WC grains that interact with oxygen. In addition, WC grains may also undergo degradation in oxygen-deficient environments, such as those located in the interior or core of the particles, according to the following reaction:

2WC ⟷ W_2_C + C (7)

It should be noted that, despite the presence of oxygen in the flame, complete oxidation is not typically achieved under HVOF conditions, as the extremely short particle residence time, locally reducing conditions, and diffusion limitations favor partial reactions such as WC decomposition and carbon depletion rather than full conversion to stable oxides, as widely reported in the literature [55,56,57]. W_2_C can form through two distinct mechanisms [58,59]. In the first case, it is produced by the decomposition of WC grains via the previously described reactions (4) and (7). In the second case, W_2_C forms during solidification of the binder phase containing Co, leading to the precipitation of W_2_C at the WC grain boundaries in the form of a spheroidal rim. Because of these reactions, part of the carbon dissolves into the metallic matrix. Once dissolved in the melt, a fraction of the carbon diffuses and reacts with oxygen to form CO and CO_2_, resulting in a net loss of carbon relative to its initial concentration in the powder. The remaining carbon in the matrix, together with W, contributes to the formation of amorphous or nanocrystalline phases, as discussed previously. The crystallite size of these phases has been reported in the literature to be below 8 nm [42,51]. Depending on the extent of decarburization, elevated levels of metallic W may also accumulate at the boundaries of the solidified particles, where carbon depletion occurs because of its reaction with oxygen [59,60]. The presence of W_2_C and W phases in the conventional coating indicates that more pronounced decarburization occurred during spraying. Although nanocrystalline WC grains are often associated with increased susceptibility to carbon loss during high-temperature processing due to their higher specific surface area [61], the larger agglomerate particle size of the NP-L powder (−75 + 38 μm compared with −50 + 10 μm for the CP powder) appears to mitigate this effect by reducing heat transfer, limiting carbon diffusion, and consequently suppressing decarburization. In addition, the spraying conditions, characterized by an increased total combustion gas flow rate, further contribute to limiting decarburization. Under these conditions, particles attain higher velocities, resulting in shorter residence times within the flame and reduced peak temperatures prior to impact, thereby minimizing WC decomposition during deposition [62].

### 3.3. Effect of Powder Particle Size on HVOF-Sprayed WC-12Co Coatings

**Figure 6:**
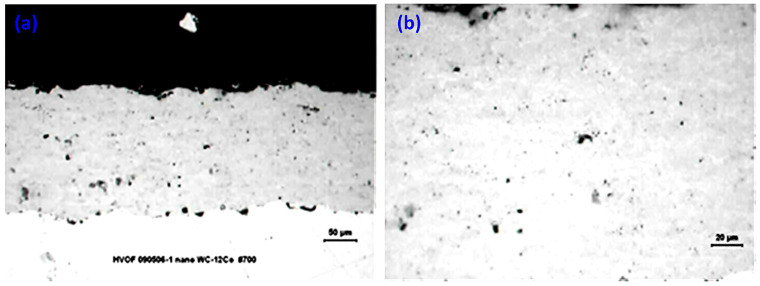
Cross-sectional OM micrographs of the nanophase coating derived from NP-S powder: (**a**) low-magnification overview; (**b**) higher-magnification detail.

The choice of particle size for the nanophase powder proved to be a key factor in defining the properties of the resulting nanophase coating, contributing to the suppression of extensive decarburization and the retention of the nanostructure after spraying. Nevertheless, the higher porosity and lower microhardness observed in comparison with the conventional coating prompted further development and investigation of a coating deposited under optimized spraying conditions. This optimization was implemented using the same nanophase WC-12Co powder, but with a reduced particle size selected to more closely approximate that of the conventional powder. As described in Section 2.1, a second nanophase WC-12Co powder (NP-S) was employed, featuring characteristics identical to NP-L but a finer particle size distribution of −38 + 15 μm (i.e., particles passing through a 38 μm sieve and retained above 15 μm). The results of the metallographic examination of the coating are presented in Figure 6 and Table 3.

For completeness and to enable direct comparison, the table also includes the properties of the conventional coating and of the nanophase coating produced using the NP-L powder, whose characteristics have been discussed previously.

The coating produced from the NP-S powder exhibits good microstructural homogeneity, absence of cracking, and an extremely low level of porosity. It also displays the characteristic lamellar structure (Figure 6a), which results from the impact and solidification of molten particles (splats) on the substrate. Overall, the micrographs reveal a strong resemblance to those of the conventional coating. Clearly, the reduction in particle size promotes more effective melting during spraying and facilitates the formation of a well-defined lamellar microstructure. The thickness per pass of the NP-S coating shows a slight improvement compared with that of NP-L, as depicted in Table 3, most likely due to the smaller particle size, which enhances particle melting and adhesion upon impact with the substrate. A pronounced reduction in porosity is observed, with porosity decreasing from 3.6% (NP-L) to 0.7% (NP-S), corresponding to an approximately 80% reduction, with values now comparable to those of the conventional coating. This behavior can be attributed to the smaller particle size, which favors more efficient deposition and reduces the formation of interparticle voids that lead to porosity. An increase in microhardness is observed for the NP-S coating, rising from 1044 HV (NP-L) to 1133 HV, accompanied by a substantial reduction in adhesion strength of approximately 40%. Notably, failure does not occur at the coating/substrate interface but rather within the coating itself, indicating low cohesive strength. This behavior constitutes a critical drawback and renders the coating unsuitable for practical application.

**Figure 7:**
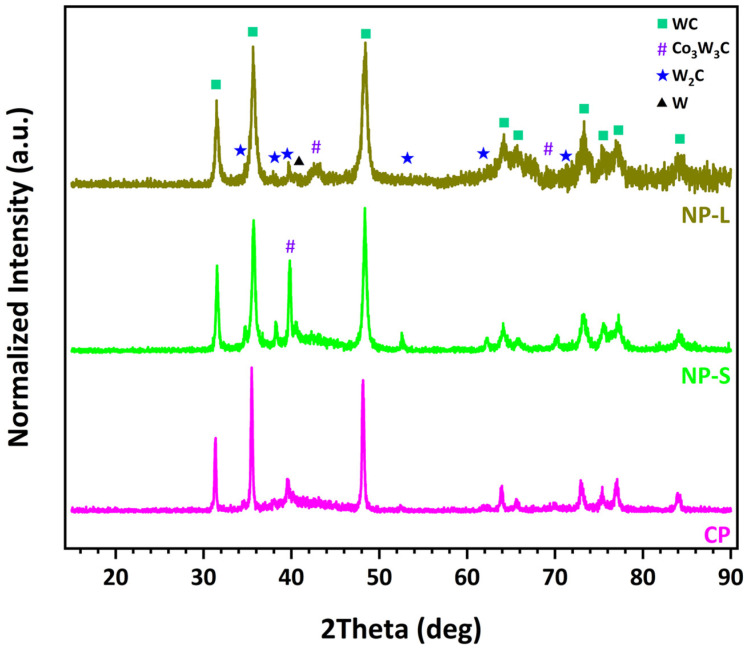
Comparative XRD patterns of HVOF-sprayed WC-12Co coatings (CP, NP-S, and NP-L), with identified crystalline phases indicated.

XRD analysis reveals features indicative of extensive decarburization in the NP-S coating (Figure 7). The detected phases are primarily WC (hexagonal), with secondary phases such as W_2_C (hexagonal) and Co_3_W_3_C (η-phase, complex cubic) depending on the processing conditions. In particular, the diffraction pattern of the NP-S coating more closely resembles that of the conventional coating than that of NP-L. The presence of the W_2_C phase is more pronounced than even in the conventional coating, and the amorphous phase in the 42–48° range is also intensified. The smaller particle size, combined with the fine WC grain size of the powder, promotes faster and more extensive degradation of the WC phase due to the high temperatures developed during spraying. These findings confirm the validity of the initial decision to employ a high combustion gas flow rate together with larger particle sizes (−75 + 38 μm) in order to achieve better retention of the nanostructure and to avoid severe degradation of the coating.

### 3.4. Effect of Aluminum Addition on HVOF-Sprayed WC-12Co Coatings

**Figure 8:**
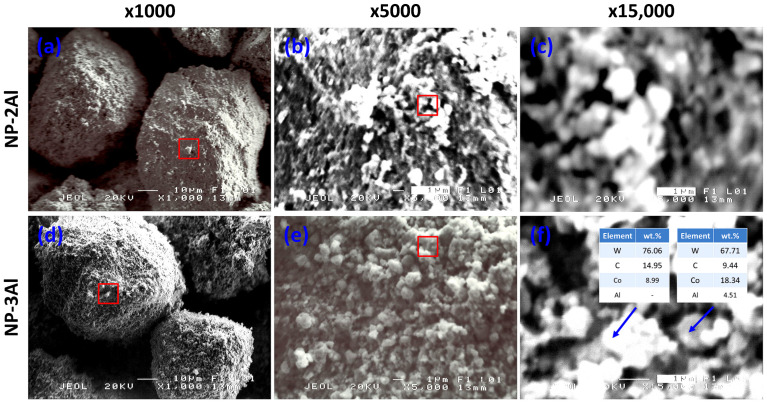
SEM micrographs of aluminum-modified nanophase powders: (**a**–**c**) NP-2Al and (**d**–**f**) NP-3Al at different magnifications. Red boxes in the lower-magnification images indicate the exact regions examined at higher magnifications. Insets in (**f**) show representative EDS point analyses with corresponding elemental compositions (wt.%).

Aluminum was incorporated to assess its potential contribution to reducing porosity and improving the cohesion of the nanophase coatings. Due to its relatively low melting point, aluminum is expected to fully melt during spraying, facilitating enhanced pore filling and improved bonding across interlamellar voids formed during the deposition process [25]. Observation of the micrographs (Figure 8) reveals no significant differences in morphology or particle shape, either among the examined powders or in comparison with the nanophase NP-L powder (Figure 2b). The representative elemental analysis of the nanophase NP-3Al powder (Figure 8f) indicates that the bright regions correspond to WC grains, whereas the gray regions are rich in Co, representing the metallic binder phase, within which WC and Al phases are also dispersed. The presence of WC within the metallic phase is attributed to the powder production route, namely high-energy ball milling, which promotes intimate mixing of the two phases through the incorporation of the hard WC phase into the softer cobalt matrix.

**Figure 9:**
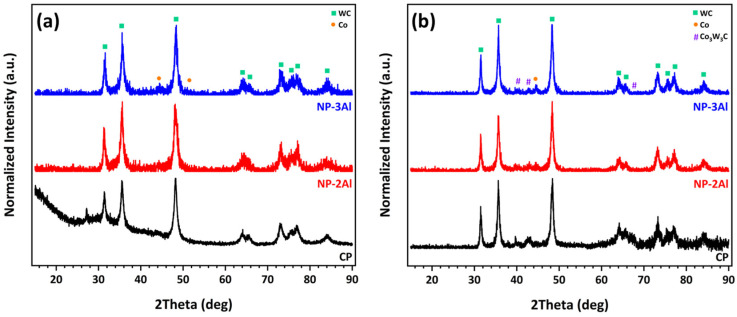
Comparative XRD patterns of (**a**) NP-L, NP-2Al, and NP-3Al powders and (**b**) their corresponding HVOF-sprayed coatings, with identified crystalline phases indicated.

Figure 9a presents a comparative XRD pattern of the three nanophase powders. The crystallite sizes of WC and Co, as estimated using the Scherrer equation, were found to range from 11–19 nm and 15–20 nm, respectively, in good agreement with the nominal crystallite sizes reported by the powder manufacturer. TEM observations [25] further indicate the actual grain size may be slightly larger than that estimated by XRD, primarily due to the possible influence of internal stresses within the crystallites, which can contribute to peak broadening. Nevertheless, in all cases, the grain size remains below 50 nm.

**Figure 10:**
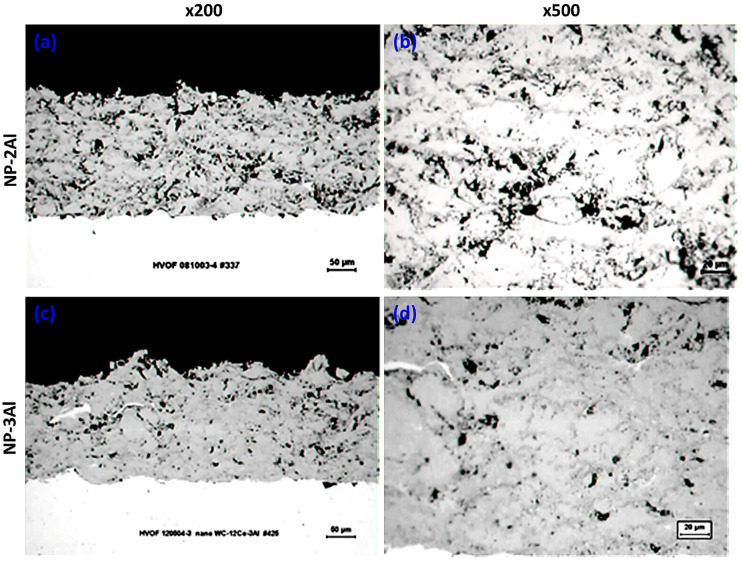
Cross-sectional OM micrographs of HVOF-sprayed nanophase coatings: (**a**,**b**) NP-2Al and (**c**,**d**) NP-3Al at different magnifications.

Representative metallographic observations of the NP-2Al and NP-3Al coatings obtained by OM are illustrated in Figure 10.

The mechanical properties and porosity of nanophase coatings are quantitatively summarized in Table 4. An examination of the data indicates that the incorporation of aluminum leads to an increase in coating microhardness, while a reduction in porosity is observed for the coating containing 3 wt.% Al. This decrease in porosity is likely associated with the low melting point of aluminum, which facilitates the filling of pores generated during spraying, particularly those related to the relatively large powder particle size. However, no systematic and clear relationship can be established between the aluminum content and either microhardness or porosity. Furthermore, the addition of aluminum does not result in any substantial variation in thickness per pass or adhesion strength.

Figure 9b presents a comparative XRD analysis of the three nanophase coatings. The diffraction patterns demonstrate that the primary WC phase is preserved in all coatings. Notably, the absence of the undesired W_2_C phase indicates that extensive decarburization of the feedstock powders has been effectively suppressed during spraying. The crystallite sizes of WC and Co, estimated using the Scherrer equation, range from 21 to 48 nm and from 11 to 18 nm, respectively. These estimates are corroborated by TEM observations, which reveal WC grain sizes of 18–28 nm and Co grain sizes of 7–12 nm [25,26]. An important finding is that the incorporation and increasing concentration of aluminum lead to a reduction in the fraction of the Co_3_W_3_C phase, which, as previously discussed, forms through the dissolution of WC grains into the molten cobalt binder during spraying. Concurrently, the emergence of distinct Co diffraction peaks in the Al-containing coatings suggests that the presence of aluminum effectively suppresses this dissolution process. This interpretation is further supported by TEM analyses and elemental mapping of the nanophase NP-2Al coating [26], which reveal that aluminum is preferentially distributed around WC grains, promoting partial separation of the WC phase from the metallic binder. A reduced extent of WC dissolution favors improved retention of the primary WC phase, which likely contributes to the observed enhancement in microhardness. The observed influence of aluminum addition can be attributed to multiple synergistic mechanisms. In particular, the low melting point of aluminum promotes enhanced densification and improved interlamellar bonding, while its presence at WC/Co interfaces appears to limit WC dissolution and suppress Co_3_W_3_C formation. At the same time, the resulting increase in hardness enhances resistance to deformation but also intensifies abrasive interactions during sliding, leading to increased friction and counterbody wear.

### 3.5. Tribological Characterization of Coatings

The tribological behavior of the investigated HVOF-sprayed WC-12Co-based coatings was systematically evaluated through long-duration pin-on-disk tests to assess frictional response, wear resistance, and dominant wear mechanisms. Specimens with dimensions of 25 × 25 mm obtained from the optimized coatings were used for the tribological evaluation. Prior to testing, surface roughness measurements were performed on the as-sprayed coatings, and the corresponding results are summarized in Table 5.

The results indicate that the CP coating in the as-sprayed condition exhibited markedly lower surface roughness compared with the coatings produced from nanophase powders, irrespective of aluminum addition, a behavior primarily attributed to the smaller particle size of the conventional feedstock, as finer particles generally lead to a more uniform surface topography after spraying. Due to these pronounced differences, the surfaces of the latter were subsequently polished in order to achieve comparable roughness levels across all samples, thereby ensuring a reliable comparison of the tribological performance. The surface preparation procedure involved initial grinding with 240-grit SiC paper, followed by sequential polishing using diamond suspensions of 45 μm, 15 μm, 9 μm, and 3 μm. After polishing, all samples were cleaned with ethanol and surface roughness measurements were repeated (Table 5). Despite extensive polishing, the surface roughness could not be reduced below 0.80 μm, as recommended by the ASTM G99-90 standard, owing to the exceptionally high resistance of coatings to material removal. Following surface preparation, long-duration pin-on-disk tests were conducted under identical and particularly severe conditions for all coatings, involving 150,000 rotations, corresponding to a total sliding distance of approximately 7070 m. Preliminary tests performed at shorter durations (30,000 and 60,000 rotations) resulted in undetectable wear of the coatings, justifying the extended test protocol adopted in this study.

**Figure 11:**
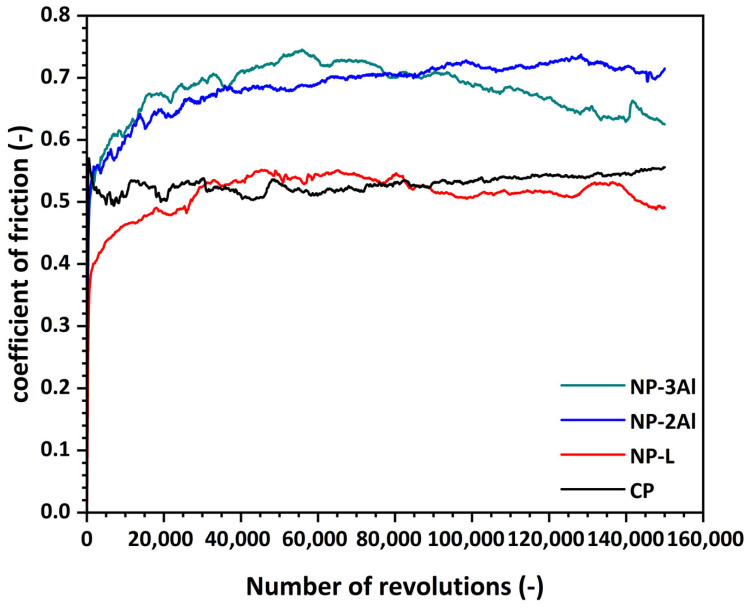
Evolution of the coefficient of friction as a function of the number of revolutions for HVOF-sprayed coatings (CP, NP-L, NP-2Al, and NP-3Al).

The NP-L coating exhibited a remarkably stable coefficient of friction throughout the entire duration of the test, with only minor fluctuations around an average value of approximately 0.51 (Figure 11). A similar frictional response was observed for the CP coating, which stabilized at an average coefficient of friction of around 0.53. In contrast, the addition of aluminum led to a pronounced increase in friction. The nanophase NP-2Al and NP-3Al coatings exhibited average coefficients of friction of approximately 0.69 and 0.68, respectively, corresponding to an increase of about 33% compared with the Al-free coatings, while counterbody wear volume increases by up to approximately sevenfold relative to the NP-L coating.

In terms of wear behavior, none of the coatings exhibited measurable wear when assessed by profilometry, even after the full test duration, indicating their exceptional resistance to sliding wear. Across all coatings, the dominant wear mechanism was identified as abrasive wear, as evidenced by the presence of dense, parallel grooves aligned with the sliding direction on the wear track surfaces. Although surface profile measurements failed to reveal clearly defined wear tracks on the coating surfaces, microscopic examination enabled qualitative and quantitative assessment of wear. Shallow wear tracks were observed, with widths varying among the different coatings. The nanophase NP-L coating exhibited the narrowest wear track, with a width of approximately 430 μm, followed closely by the nanophase NP-2Al coating at approximately 440 μm. The wear track width increased to about 650 μm for the NP-3Al coating, indicating a slightly reduced wear resistance. The conventional CP coating exhibited a wear track width of approximately 470 μm, comparable to that of the nanophase NP-L coating.

Distinct differences were observed in the degree of aggressiveness of the coatings toward the counterbody material. The nanophase NP-L coating induced the lowest counterbody wear, with a wear track width of approximately 700 μm. The incorporation of aluminum resulted in a pronounced increase in counterbody wear: for the NP-2Al coating, the wear track width reached approximately 1140 μm, whereas for the NP-3Al coating it decreased to about 960 μm, representing an intermediate level between the Al-free nanophase coating and the 2 wt.% Al variant. The conventional CP coating produced the most severe counterbody wear, exhibiting a wear track width of approximately 1170 μm, the highest among all coatings examined. These results indicate that, despite the excellent intrinsic wear resistance of all coatings, variations in coating properties strongly influence the extent of wear imposed on the opposing material.

Given the negligible wear observed on the coating surfaces, counterbody wear was quantified in accordance with the ASTM G99-90 standard, which enables reliable estimation of volume loss when wear of the disk specimen is insignificant. In this approach, the counterbody (spherical ball) volume loss, V_loss_ (mm^3^), was calculated from the measured wear scar diameter, D (mm), using the following standard geometric relationship [30,63]:

(8) $$V_{loss}=\frac{\pi \times D^{4}}{64\times R}$$

where R is the radius of the counterbody (mm). The calculated values are summarized in Table 6. In this table, w denotes the wear track width of the coating (μm), while w_c_ corresponds to the wear track width of the counterbody material (μm).

The comparative results reveal a strong correlation between coating microhardness and counterbody wear. The nanophase NP-L coating, which exhibits the lowest microhardness among the tested coatings, induces the smallest counterbody wear track width (approximately 694 μm) and the lowest volume loss (0.0038 mm^3^). In contrast, coatings with higher microhardness generated wider wear tracks and substantially higher volume losses. The addition of aluminum significantly increases counterbody wear, with the NP-2Al coating exhibiting the largest volume loss (0.0269 mm^3^), despite maintaining excellent intrinsic wear resistance. The NP-3Al coating shows intermediate behavior, while the CP coating produces the most severe counterbody wear overall, characterized by the widest wear track (approximately 1170 μm) and the highest volume loss (about 0.0305 mm^3^). These findings confirm that increased coating microhardness enhances abrasive interaction with the opposing material during sliding contact, leading to more pronounced counterbody wear even when coating wear remains negligible. This behavior contrasts with previous studies reporting improved wear resistance in nanostructured WC-Co coatings, where increased hardness is typically associated with enhanced performance, thereby highlighting the critical role of processing-induced phase stability and the importance of evaluating counterbody wear in tribological systems.

Overall, all HVOF-sprayed WC-12Co-based coatings demonstrate outstanding resistance to sliding wear under severe testing conditions, with coating wear remaining negligible even after extended sliding distances. However, the comparative analysis highlights important differences in frictional behavior and counterbody wear. The nanophase NP-L coating without aluminum addition provides the most balanced tribological performance, combining low friction with minimal damage to the counterbody, whereas aluminum additions, although beneficial for certain mechanical properties, result in increased friction and more severe counterbody wear.

## 4. Discussion

In the optimized coating, the coexistence of grains with sizes of only a few nanometers alongside grains in the range of 1–2 μm was observed, the latter likely associated with agglomeration phenomena. For this reason, the coating can be classified as nanophase rather than fully nanocrystalline. Microstructural analysis revealed that the initial metallic cobalt phase is largely transformed into Co_3_W_3_C after spraying. In contrast, the conventional coating exhibits the presence of W_2_C and W phases, indicative of pronounced decarburization during deposition. Furthermore, the original Co phase is not detected in the conventional coating, while the formation of an amorphous phase is observed. The particle size of the feedstock powder plays a critical role in determining both the resulting microstructure and properties of the coatings. A reduction in particle size intensifies decarburization phenomena during spraying, leading to coatings that increasingly resemble the conventional counterpart in both structure and behavior. These observations are consistent with previously reported decarburization mechanisms in HVOF-sprayed WC-Co coatings, where limited oxygen diffusion and short residence times favor partial reactions such as WC decomposition and W_2_C formation rather than complete oxidation [54,55,56].

The addition of Al (2–3 wt.%) increases microhardness and reduces porosity, leading to a microhardness increase of up to 16% while inducing up to a sevenfold increase in counterbody wear, although no systematic correlation with aluminum content is observed. In addition, the presence of aluminum effectively suppresses the dissolution of WC into the cobalt binder and limits the formation of the Co_3_W_3_C phase, thereby contributing to improved retention of the primary WC phase. Tribological evaluation indicates that both conventional and nanophase WC-12Co coatings exhibit comparable coefficients of friction, whereas the addition of aluminum results in an increase in friction. The wear of the coatings themselves was negligible and could not be quantified, highlighting their exceptional resistance to sliding wear under the applied test conditions. With respect to the counterbody material, the nanophase WC-12Co coating induces the lowest wear, while the conventional coating causes the highest. A linear correlation is identified between counterbody wear and coating microhardness, and all coatings primarily exhibit abrasive wear mechanisms. The observed variations in coating thickness, morphology, phase composition, and porosity are directly linked to the thermal and kinetic conditions experienced during HVOF spraying. In particular, differences in particle size and composition influence heat transfer, degree of melting, and WC dissolution, thereby governing phase stability and microstructural evolution during deposition. These combined effects ultimately determine the resulting mechanical and tribological performance of the coatings.

These findings highlight the critical importance of carefully controlling feedstock characteristics and spray parameters in order to achieve an optimal balance between microstructural stability and tribological performance of WC-Co-based coatings. In particular, precise adjustment of powder particle size, chemical composition, and spraying conditions is essential to minimize undesirable phase transformations and preserve the integrity of the hard WC phase. Overall, the results confirm that coating performance is governed by the interplay between phase evolution and tribological interactions, rather than by individual properties alone. Future research may focus on extending the present analysis to fretting and sliding conditions at elevated temperatures, where thermally activated degradation mechanisms become more pronounced. In addition, systematic optimization of aluminum additions, in combination with tailored spraying parameters, could further enhance coating performance and facilitate the development of application-specific solutions for demanding industrial environments. A broader compositional range of aluminum addition may further elucidate the transition between beneficial and detrimental tribological effects and support the design of optimized compositions.

## 5. Conclusions

The results of this study demonstrate that feedstock particle size is a governing factor for microstructural stability and overall performance of HVOF-sprayed nanophase WC-12Co coatings. The use of the larger nanophase powder enabled effective retention of nanoscale WC features while mitigating extensive decarburization during spraying, resulting in coatings with good adhesion strength and acceptable porosity. In contrast, reducing the particle size, although beneficial for improving deposition efficiency, reducing porosity, and increasing microhardness, promoted more severe phase degradation and led to a substantial loss of cohesive integrity. This degradation was evidenced by a ~40% decrease in adhesion strength, with failure occurring within the coating rather than at the coating/substrate interface, rendering this condition unsuitable for practical application despite its favorable hardness and density.

Aluminum additions in the range of 2–3 wt.% further influenced coating performance by enhancing microhardness and reducing the extent of WC dissolution into the Co binder, as demonstrated by a lower contribution of the Co_3_W_3_C phase and improved preservation of the primary WC phase. These benefits were achieved without strongly affecting deposition efficiency or adhesion strength. Under severe ASTM G99 sliding conditions, all coatings exhibited negligible disk wear, and abrasive wear was identified as the dominant mechanism, confirming their excellent intrinsic wear resistance. Nevertheless, aluminum additions led to increased friction coefficients and more pronounced counterbody wear. A clear correlation between coating microhardness and counterbody wear was established, highlighting a critical trade-off between enhancing coating hardness and minimizing wear to the opposing material in tribological contacts. Overall, the results demonstrate that optimizing WC-Co coatings requires balancing competing mechanisms including minimizing decarburization while maintaining sufficient cohesion and controlling counterbody wear. In particular, increases in hardness and densification may lead to up to a sevenfold increase in counterbody wear, highlighting a key limitation that is not commonly addressed in previous studies and emphasizing the need for system-level tribological optimization rather than property-based optimization alone.

From an application perspective, the nanophase WC-12Co coating without aluminum addition (NP-L) provides the most balanced tribological performance and is therefore particularly suitable for sliding systems where minimal counterbody damage is required, such as precision mechanical components, hydraulic systems, and wear-sensitive interfaces. In contrast, coatings with higher microhardness, including Al-modified and conventional WC-Co coatings, may be more appropriate for applications where maximum hardness and load-bearing capacity are prioritized over counterbody preservation, such as cutting and forming tools.

## Figures and Tables

**Table 1:** HVOF spraying parameters applied for the deposition of WC-Co coatings.

Sample	Distance	O_2_/Fuel Ratio	Particle Velocity	Feed Rate
	(mm)	(-)	(mm/s)	(g/min)
NP-L, NP-S	200	4.69	1200	50
CP	200	5.12	1000	38

**Table 2:** Microstructural and mechanical properties of optimized WC-12Co coatings.

Sample	Thickness Per Pass, t_p_	Microhardness	Adhesion Strength, σ_adh_	Porosity, P
	(μm)	(HV_0.3_)	(MPa)	(%)
NP-L	9.3 ± 1.3	1044 ± 187	60.0 ± 2.4	3.6 ± 1.1
CP	7.5 ± 0.3	1208 ± 62	64.8 ± 4.9	0.5 ± 0.1

**Table 3:** Microstructural and mechanical properties of HVOF-sprayed WC-12Co coatings produced from NP-S, NP-L, and CP powders.

Sample	t_p_	Microhardness	σ_adh_	P
	(μm)	(HV_0.3_)	(MPa)	(%)
NP-L	9.3 ± 1.3	1044 ± 187	60.0 ± 2.4	3.6 ± 1.1
NP-S	10.3 ± 0.4	1133 ± 109	36.3 ± 1.8	0.7 ± 0.2
CP	7.5 ± 0.3	1208 ± 62	64.8 ± 4.9	0.5 ± 0.1

**Table 4:** Microstructural and mechanical properties of HVOF-sprayed coatings produced from nanophase powders.

Sample	t_p_	Microhardness	σ_adh_	P
	(μm)	(HV_0.3_)	(MPa)	(%)
NP-L	9.3 ± 1.3	1044 ± 187	60.0 ± 2.4	3.6 ± 1.1
NP-2Al	10.0 ± 1.0	1215 ± 159	60.2 ± 2.7	3.8 ± 0.8
NP-3Al	9.6 ± 1.0	1178 ± 202	60.2 ± 2.1	2.8 ± 0.9

**Table 5:** Surface roughness (Ra) of the coatings after spraying and prior to tribological testing.

Sample	Ra After Spraying (μm)	Ra Prior to Tribological Testing (μm)	Average Ra (μm)
NP-L	8.50–11.25	2.62–5.17	3.97
NP-2Al	8.60–12.70	3.47–5.76	4.68
NP-3Al	9.35–12.33	2.41–3.92	3.07
CP	2.16–11.25	2.26–2.74	2.48

**Table 6:** Summary of tribological performance and counterbody wear of HVOF-sprayed WC-12Co coatings.

Sample	μ (Avg.)	w	w_c_	V_loss_	Microhardness
	(-)	(μm)	(μm)	(mm^3^)	(HV_0.3_)
NP-L	0.51	430	694.3	0.003802	1044
NP-2Al	0.69	440	1132.4	0.026906	1215
NP-3Al	0.68	650	964.0	0.014130	1178
CP	0.53	470	1170.0	0.030484	1208

## Data Availability

The original contributions presented in this study are included in the article. Further inquiries can be directed to the corresponding authors.
